# Prevalence, characteristics and challenges of late HIV diagnosis in Germany: an expert narrative review

**DOI:** 10.1007/s15010-023-02064-1

**Published:** 2023-07-20

**Authors:** Christoph Boesecke, Sven Schellberg, Jochen Schneider, Gundolf Schuettfort, Hartmut Stocker

**Affiliations:** 1 Department of Medicine, University Hospital Bonn, Bonn, Germany; 2 Novopraxis Berlin GbR, Berlin, Germany; 3 School of Medicine, University Hospital Rechts der Isar, Technical University of Munich, Munich, Germany; 4 Department of Infectious Diseases, University Hospital Frankfurt, Frankfurt, Germany; 5 Department of Infectious Diseases, St. Joseph Hospital, Berlin, Germany

**Keywords:** Late HIV diagnosis, Germany, COVID-19, Ukraine war, Universal testing, Indicator testing, ART

## Abstract

**Purpose:**

We aimed to review the landscape of late HIV diagnosis in Germany and discuss persisting and emerging barriers to earlier diagnosis alongside potential solutions.

**Methods:**

We searched PubMed for studies informing the prevalence, trends, and factors associated with late HIV diagnosis in Germany. Author opinions were considered alongside relevant data.

**Results:**

In Germany, older individuals, heterosexuals, and migrants living with HIV are more likely to be diagnosed late. The rate of late diagnosis in men who have sex with men (MSM), however, continues to decrease. Indicator conditions less often prompt HIV testing in women and non-MSM. During the COVID-19 pandemic, the absolute number of late diagnoses fell in Germany, but the overall proportion increased, probably reflecting lower HIV testing rates. The Ukraine war and subsequent influx of Ukrainians living with HIV may have substantially increased undiagnosed HIV cases in Germany. Improved indicator testing (based on unbiased assessments of patient risk) and universal testing could help reduce late diagnoses. In patients who receive a late HIV diagnosis, rapid treatment initiation with robust ART regimens, and management and prevention of opportunistic infections, are recommended owing to severely compromised immunity and increased risks of morbidity and mortality.

**Conclusion:**

Joint efforts are needed to ensure that UNAIDS 95-95-95 2030 goals are met in Germany. These include greater political will, increased funding of education and testing campaigns (from government institutions and the pharmaceutical industry), continued education about HIV testing by HIV experts, and broad testing support for physicians not routinely involved in HIV care.

## Introduction

The past 40 years have seen significant advancements in human immunodeficiency virus (HIV) treatment, with most people living with HIV (PLWH) now able to lead normal, healthy lives with the help of modern single-tablet regimens (STRs). Given that virologic suppression has been shown to prevent transmission, and that pre-exposure prophylaxis (PrEP) can prevent infection, ending the HIV epidemic is not just a possibility, but a realistic goal [1]. To work toward this objective, the World Health Organization has set the 95-95-95 target (95% PLWH diagnosed, of whom 95% are on antiretroviral therapy [ART], of whom 95% are virologically suppressed) to be achieved by 2030 [2]. However, a major threat to this goal is late diagnosis, defined as diagnosis when the CD4 count is < 350 cells/µL or the patient presents with an acquired immune deficiency syndrome (AIDS)-defining event [3]. A proportion of these patients will have advanced HIV disease at diagnosis (defined as a CD4 count < 200 cells/µL or clinical AIDS) [3]. In 2022, the definition for late diagnosis was revised to ensure that people with evidence of recent infection (either by laboratory evidence, a last negative HIV test within 12 months of diagnosis, or clinical evidence of acute infection) and a corresponding drop in CD4 cell counts in the acute infection phase are not included in the statistics for a late diagnosis [4]. This is especially important in settings with a high frequency of HIV testing (e.g., testing associated with PrEP, self-testing opportunities, and low-barrier testing programs).

**Fig. 1:**
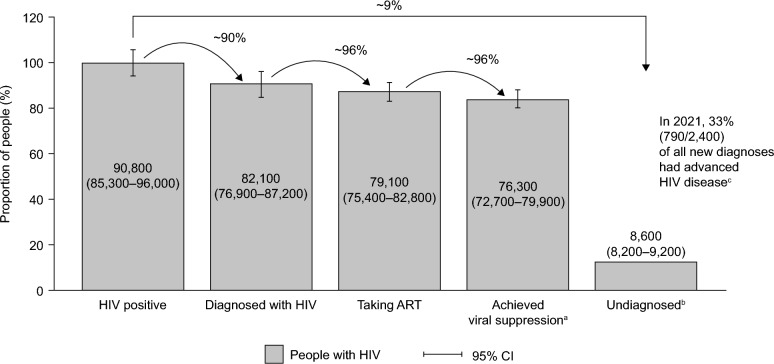
HIV statistics across the treatment cascade in Germany in 2021, including the estimated number of people with HIV who are unaware of their diagnosis and the proportion of late diagnoses (adapted with permission from the RKI) [5]. ^a^viral load < 200 copies/mL. ^b^Data does not include people infected with HIV abroad who now live in Germany with undiagnosed HIV. ^c^AIDS or CD4 count < 200 cells/µL. *AIDS* acquired immunodeficiency syndrome, *CI* confidence interval, *HIV* human immunodeficiency virus, *RKI* Robert Koch Institute

In Germany, the rates of linkage to ART and successful ART are high (2021: 96% and 96%, respectively). However, the rate of HIV diagnosis (2021: 90%) is somewhat below what would be expected [5]. Therefore, as with other European countries, there remains a significant number of PLWH who are unaware of their infection or receive a late diagnosis, with little improvement in these statistics observed in recent years (Fig. 1) [6, 7].

Late diagnosis is associated with increased risks of HIV transmission and treatment failure (with the subsequent development of resistance), and increased risks of clinical progression, treatment-related adverse events, opportunistic infections, non-infectious comorbidities, mortality, and multi-morbidity, and thus imposes substantial societal and economic costs [6, 8–11].

In this review, we summarize evidence on the status of late HIV diagnosis in Germany, including its prevalence, associated patient characteristics, and current recommendations for treatment, as well as the impact of events, such as the COVID-19 pandemic and the war in Ukraine. Most importantly, we highlight what still needs to be addressed in Germany to achieve further reductions in persisting rates of late HIV diagnosis to ensure resilience to emerging and future challenges.

## Prevalence and characteristics of late HIV diagnosis in Germany

### Prevalence of late diagnosis

The prevalence and characteristics of late HIV diagnosis in Germany were first investigated using national case surveillance data from the Robert Koch Institute (RKI, a federal government agency and research institute responsible for disease control and prevention) (2001–2010; *N* = 22,925), and data from the Clinical Surveillance of HIV Disease (ClinSurv) cohort (1999–2010; *N* = 6897) [12]. Prevalence estimates were reported at 49.5% for late HIV diagnosis and 58.1% for late HIV presentation for care (defined as having a CD4 count < 350 cells/μL or clinical AIDS at the first contact at a treatment center participating in the ClinSurv cohort) [12]. Similar to other European countries [13], the prevalence of late HIV diagnosis in Germany has remained high in the past decade. Data from HIV-Regional (2014), covering 31 HIV-focused centers (15 hospital outpatient clinics and 16 medical practices) reported late HIV diagnosis in 55.6% of 971 patients, with 33.5% having advanced HIV disease [3, 14]. Data from the national statutory notification of newly diagnosed HIV infections (2011–2018) reported diagnosis with a non-recent HIV infection (defined as an infection diagnosed after a mean duration of more than five months since it was acquired [as determined by the BED-Capture-ELISA]) in 67.5% of 16,010 patients, with 15.2% (2746/18,092) diagnosed with AIDS [15]. These findings are consistent with the most recent national surveillance report showing that 33.0% of new diagnoses in 2021 were for advanced HIV disease (Fig. 1) [5]. Taken together, the available data indicate that the rate of late HIV diagnosis in Germany has not changed markedly in the past two decades, with around one-third of diagnoses presenting with severe immunodeficiency.

### Late diagnosis—patient characteristics

**Fig. 2:**
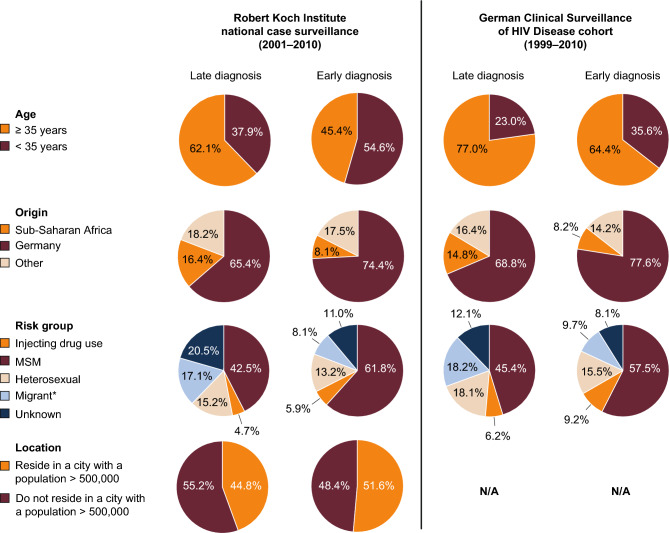
Characteristics of patients with a late HIV diagnosis in Germany from 1999 to 2010 (adapted with permission from Zoufaly et al. [12]). Late diagnosis was defined as a CD4 cell count < 350 cells/µL or clinical AIDS; early diagnosis was defined as a CD4 cell count ≥ 350 cells/µL without clinical AIDS.*CD4 cell counts were missing for 73% of patients sourced from the RKI and were more often reported for individuals with poor clinical status. Missing CD4 data were thus imputed to avoid overestimating the proportion of late HIV diagnoses. This was achieved by estimating probabilities of patients having a high (≥ 350 cells/µL) versus low (< 350 cells/µL) CD4 cell count based on diagnosis age and date, transmission risk, and residence in big cities. *HIV* human immunodeficiency virus, *MSM* men who have sex with men, *NA* not available, *RKI* Robert Koch Institute

Both RKI (2001–2010) and ClinSurv (1999–2010) data indicated that older individuals (≥ 35 years), heterosexuals, and individuals from sub-Saharan Africa were significantly over-represented among patients who are diagnosed with HIV late versus early (defined as a CD4 cell count ≥ 350 cells/μL without clinical AIDS) in Germany, while men who have sex with men (MSM), individuals who inject drugs, and those located in big cities were under-represented (Fig. 2) [12].

Further analysis of these data showed that, compared with MSM, the probability of being diagnosed late was significantly higher for heterosexuals (odds ratios [ORs] of 1.5 and 1.4 for respective data sources), individuals with a history of migration from countries with a high (> 1.0%) HIV prevalence (ORs of 2.9 and 2.1), and patients with unknown risk (ORs of 2.2 and 1.5) [12]. Within the heterosexual and migrant populations, women had a lower probability of late diagnosis compared with men, with the authors concluding this was probably due to effective antenatal testing (ORs for the respective data sources, heterosexuals: 0.65 and 0.59; migrants: 0.74 and 0.72) [12]. More recent data (2009–2013) from a retrospective single cohort study in Berlin showed that women were significantly more likely to present with an indicator condition (IC) and not be tested for HIV compared with men (OR 4.7), perhaps driven by the belief that women are not at risk of HIV infection [16]. Perhaps driven by the same belief, non-MSM were also more likely to present with an IC and not be tested for HIV compared with MSM (OR 2.4) in this analysis.

Regional socioeconomic status also affects the likelihood of late diagnosis, but only for certain population groups. Data from the national statutory notification of newly diagnosed HIV infections (2011–2018) showed that MSM living in highly deprived regions in the countryside were more likely to be diagnosed late than those living in less-deprived areas [15]. However, this difference was not observed in MSM from towns or major cities. Although individuals with heterosexual contact were generally at a higher risk of being diagnosed late with HIV compared with MSM, for heterosexuals, this risk was unrelated to their socioeconomic status [15]. A retrospective study of people with HIV in Dresden, Germany (1986–2010), identified a positive syphilis screening test in MSM as a significant determinant for lower risks of late diagnosis, suggesting that HIV prevention strategies may benefit from a focus on the diagnosis of other (often co-occurring) sexually transmitted infections [17].

In conclusion, these studies highlight the range of health and social factors that affect rates of late diagnosis and suggest that multiple targeted approaches will probably be required to address this problem in Germany, rather than a one-size-fits-all approach.

### Longitudinal changes in patient characteristics

**Fig. 3:**
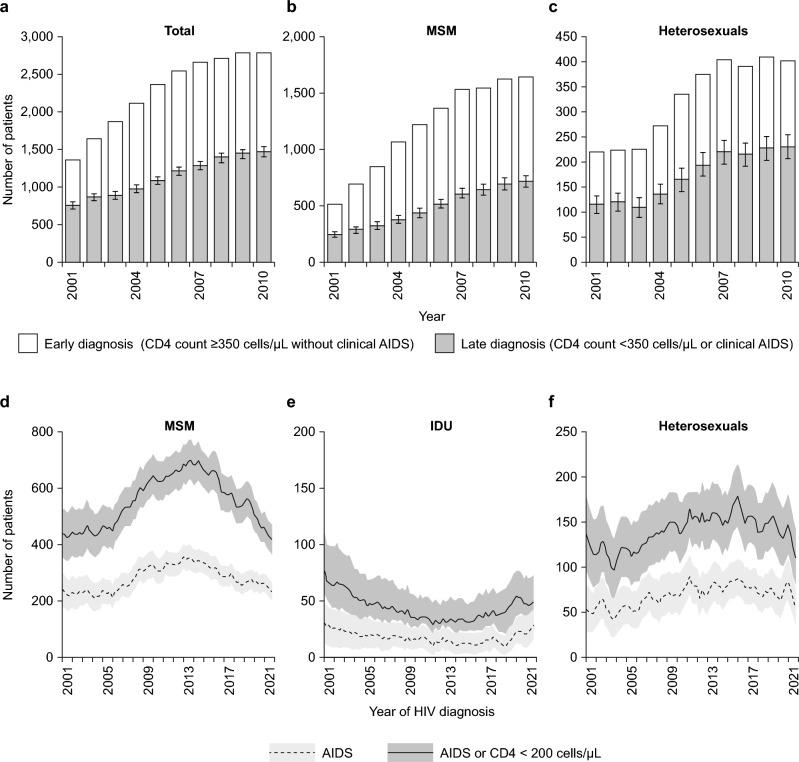
Changes in characteristics of individuals diagnosed late with HIV in Germany from 2001 to 2021. Reproduced with permission from Zoufaly et al. (panels a, b and c) [12] and the RKI (panels d–f) [5]. *HIV* human immunodeficiency virus, *IDU* injectable drug use, *MSM* men who have sex with men, *RKI* Robert Koch Institute

Although the overall proportion of individuals who are diagnosed late with HIV in Germany has not changed considerably over time, fluctuations in the characteristics of different patient groups have been observed. ClinSurv data suggest that, in Germany, the proportion of late HIV diagnoses both overall and among heterosexuals did not change markedly from 2001 to 2010, while the proportion attributed to MSM decreased (Fig. 3a, b and c) [12]. Data from the RKI epidemiological bulletin show a steady reduction in the absolute numbers of MSM with advanced HIV disease since 2013 (Fig. 3d), whereas the numbers of heterosexuals and injection drug users diagnosed with advanced disease have changed little in this time. However, in the last few years, the number of heterosexuals diagnosed with advanced HIV disease has started to decline, in contrast to the slight increase seen among injection drug users (Fig. 3e and f) [5]. It is important to acknowledge, however, that a decrease in the proportion of new HIV diagnoses that are late can occur even if the absolute number of late diagnoses increases. Indeed, this was the case for MSM between 2000 and 2010, when the proportion of late HIV diagnoses decreased, but the absolute number of late and advanced HIV diagnoses increased (Fig. 3b and d). Therefore, the rate of late HIV diagnoses should always be considered alongside the total number of new infections when informing overall strategies for HIV management.

### Clinical presentation of patients with a late HIV diagnosis

**Fig. 4:**
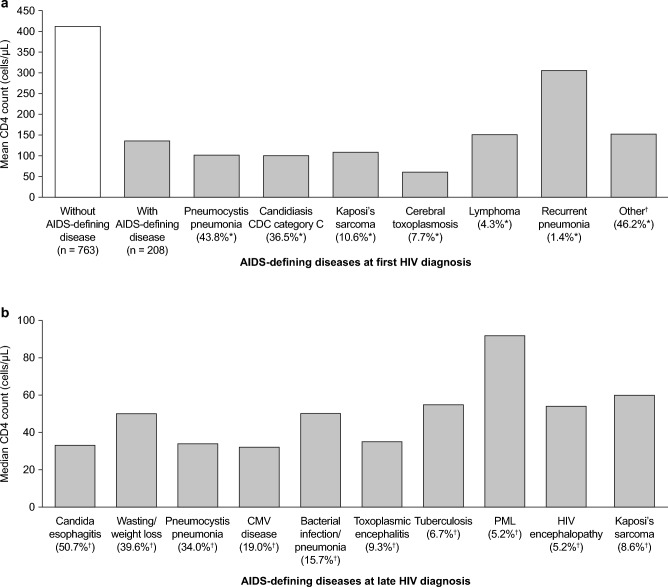
Most common AIDS-defining diseases in Germany and associated CD4 counts. Panel a shows 2014 data from individuals with a first HIV diagnosis (adapted with permission from Table 3 in Schleenvoigt et al.) [14] and panel b shows 2009–2013 data from patients with a late HIV diagnosis (adapted with permission from Table 2 in Tominski et al. [16]). ^*^Proportion of individuals at first HIV diagnosis with AIDS-defining disease (*n* = 208). ^†^Proportion of individuals with a late HIV diagnosis who present with AIDS-defining disease (*n* = 268). *AIDS* acquired immunodeficiency syndrome, *CDC* Centers for Disease Control and Prevention, *CMV* cytomegalovirus, *HIV* human immunodeficiency virus, *PML* progressive multifocal leukoencephalopathy

Data from the HIV-Regional analysis confirm that in Germany, as with other countries, a low CD4 count at the time of HIV diagnosis is associated with the presence of AIDS-defining diseases (Fig. 4a), with pneumocystis pneumonia and candidiasis the most commonly identified ICs in this study [14]. These were also among the most common diseases in patients with a late HIV diagnosis in the retrospective study in Berlin, along with wasting/weight loss, cytomegalovirus disease, and bacterial infection/pneumonia (Fig. 4b) [16].

Another key finding from the Berlin study was that 20.5% (55/268) of those receiving a late diagnosis had presented to municipal healthcare services with at least one IC prior to HIV diagnosis, without being offered a test for HIV [16]. The most commonly missed ICs were leukocytopenia, thrombocytopenia, oral candidiasis, unexplained weight loss, herpes zoster (men), and cervical dysplasia (women) [16]. Notably, more than 40% of these patients with ICs presented to the emergency department (ED) [16]. As the municipal service provides medical care for only about one-third of the population of Berlin, the overall rate of prior healthcare presentation with an IC is probably much higher [16].

## Considerations for treatment of patients with a late diagnosis

### Recommendations for ART

The 2020 German–Austrian guidelines for the treatment of HIV [18] largely mirror those developed by the European AIDS Clinical Society (EACS; updated October 2022) [19], which are widely used by HIV-treating physicians in Germany. In general, both guidelines recommend combination therapies based on optimal treatment characteristics, specifically long-term efficacy, low propensity for the emergence of drug resistance or drug–drug interactions (DDIs), and safety and tolerability [18, 19]. Both guidelines highlight the need to choose initial ART according to the individual characteristics and needs of each PLWH, and specify a greater urgency to begin ART immediately for PLWH with more severe immunodeficiency (such as lower CD4 cell counts or pregnancy) [18, 19]. Although neither guideline refers specifically to ART selection for individuals with late diagnosis, both provide guidance around the selection of ART regimens for rapid treatment initiation, which would apply to most individuals with a late diagnosis [18, 19]. Rapid start is characterized by treatment initiation in the absence of baseline laboratory parameters (such as resistance test results), and several robust first-line treatment regimens are recommended for rapid start in both guidelines [18, 19]. It should be noted that the management of patients diagnosed with advanced HIV disease is a particular challenge for clinicians. Patients with more severe disease at diagnosis are at the highest risk of treatment failure and of developing resistance, yet there is a lack of clinical trial data to guide disease management (discussed in more detail below). Minimizing DDIs is particularly important in these patients owing to higher rates of concomitant treatment required for co-infections (such as hepatitis C virus or tuberculosis, opportunistic infections (OIs), and comorbidities [9, 10, 20, 21]). Management and prevention of OIs is a challenge after late diagnosis, but clinical guidelines are well-established [18, 19]. More detailed ART treatment recommendations are beyond the scope of this review; however, a summary of recommended first-line HIV treatments for ART-naive adults from the EACS guidelines—including those for rapid start—is provided in Table 1 [19].

**Table 1:** Recommended* first-line HIV treatments for ART-naive adults taken from the 2022 EACS guidelines [19]

Regimen	Main requirements	Additional guidance (see footnotes)
2 NRTIs + INSTI		
ABC/3TC + DTG ABC/3TC/DTG	HLA-B*57:01-negative HBsAg-negative	I.(ABC: HLA-B*57:01, cardiovascular risk) II.(DTG: weight increase)
TAF/FTC/BIC		II.(BIC TAF: weight increase)
TAF/FTC + DTG TDF/XTC + DTG		II. (DTG, TAF: weight increase) III. (TDF: prodrug types. Renal and bone toxicity. TAF dosing)
TAF/FTC + RAL qd or bid TDF/XTC + RAL qd or bid		II. (RAL, TAF: weight increase) III. (TDF: prodrug types. Renal and bone toxicity. TAF dosing) IV. (RAL: dosing)

1 NRTI + INSTI		
XTC + DTG 3TC/DTG	HBsAg-negative HIV-VL < 500,000 copies/mL Not recommended after PrEP failure	II. (DTG: weight increase) V. (3TC/DTG not after PrEP failure)

2 NRTIs + NNRTI		
TAF/FTC + DOR TDF/XTC + DOR TDF/3TC/DOR		II. (TAF: weight increase) III. (TDF: prodrug types. Renal and bone toxicity. TAF dosing) VI. (DOR: caveats, HIV-2)

I. ABC contraindicated if HLA-B*57:01-positive, not to be used for same day start. Even if HLA-B*57:01-negative, counseling on hypersensitivity reaction risk still mandatory. ABC should be used with caution in persons with a high CVD risk (> 10%) (page 62 of EACS guidelines)

II. Treatment with INSTIs or TAF may be associated with weight increase

III. In certain countries, TDF is labeled as 245 mg rather than 300 mg to reflect the amount of the prodrug (tenofovir disoproxil) rather than the fumarate salt (tenofovir disoproxil fumarate). There are available generic forms of TDF, which instead of fumarate use phosphate, maleate, and succinate salts. They can be used interchangeably. When available, combinations containing TDF can be replaced by the same combinations containing TAF. TAF is used at 10 mg when co-administered with drugs that inhibit P-gp, and at 25 mg when co-administered with drugs that do not inhibit P-gp

The decision whether to use TDF or TAF depends on individual characteristics as well as availability

If the ART regimen does not include a booster, TAF and TDF have a similar short-term risk of renal AEs leading to discontinuation and bone fractures

TAF*** should be considered as a first choice**** over TDF in individuals with:

Established or high risk of CKD (see page 74 of EACS guidelines)

Co-administration of medicines with nephrotoxic drugs or prior TDF toxicity (see page 75 of EACS guidelines)

Osteoporosis / progressive osteopenia, high FRAX score or risk factors (see page 71 of EACS guidelines)

History of fragility fracture (see pages 71 and 73 of EACS guidelines)

IV. RAL can be given as RAL 400 mg bid or RAL 1200 mg (two, 600 mg tablets) qd. Note: RAL qd should not be given in presence of an inducer (i.e., TB drugs, antiepileptics) or divalent cations (i.e., calcium, magnesium, iron), in which case RAL should be used bid

V HIV infections occurring in the context of PrEP failure may be associated with resistance-associated mutations. 3TC/DTG may be used in in this context only if there is no documented resistance in genotypic test

IV DOR is not active against HIV-2. DOR has not been compared to an INSTI and was shown to be non-inferior to EFV and DRV. There is risk of resistance-associated mutations in case of virological failure. Results of genotypic resistance test are necessary before starting DOR

**Recommendations for rapid start:** If therapy needs to be started before the results of resistance testing become available, preference should be given to starting a PI/b or an INSTI with high barrier to resistance (DTG or BIC), in order to increase the barrier to resistance of the overall regimen. More than three active drugs are not needed. A potential advantage for selecting DTG or BIC is the faster viral load suppression. The benefit of combining PI/b with INSTI has not been shown. A combination of TDF or TAF, FTC, and either DRV/b, DTG or BIC, should therefore be considered, and the regimen adjusted, if needed, once the resistance test becomes available and viral load suppression is achieved. Where such a regimen is not available, national epidemiological data on prevalence and patterns of transmitted drug resistance (where available and sufficiently representative) may assist with the treatment selection process [19]

*AE* adverse event, *ART* antiretroviral treatment, *ABC* abacavir, *AIDS* acquired immunodeficiency syndrome, *BIC* bictegravir, *CKD* chronic kidney disease, *CVD* cardiovascular disease, *DDI* drug-drug interaction, *DOR* doravirine, *DRV* darunavir, *DTG* dolutegravir, *EACS* European Acquired immune deficiency syndrome Clinical Society, *FRAX* Fracture Risk Assessment Tool, *FTC* emtricitabine, *HBsAg* Hepatitis B surface antigen, *INSTI* integrase strand transfer inhibitor, *HIV* human immunodeficiency virus, *HIV-2* human immunodeficiency virus type 2, *HLA-B* human leukocyte antigen, *NNRTI* non-nucleoside reverse transcriptase inhibitors, *NRTI* nucleoside reverse transcriptase inhibitors, *P-gp* P-glycoprotein, *PI/b*, protease inhibitors pharmacologically boosted with cobicistat or ritonavir, *PrEP* pre-exposure prophylaxis, *RAL* raltegravir, *3TC* lamivudine, *TAF* tenofovir alafenamide, *TB* tuberculosis, *TDF* tenofovir disoproxil fumarate, *XTC* 3TC or FTC

^*^Recommended therapies are based on combination of essential characteristics for an optimal treatment such as long-term efficacy, barrier to resistance, safety, tolerability, and few DDIs. Alternative regimens (not presented in this table) are recommended for instances in which recommended regiments are not feasible

^***^There are limited data on use of TAF with estimated glomerular filtration rate < 10 mL/min

^****^Expert opinion pending clinical data

### Comparison of ART regimens in PLWH with late HIV diagnosis

There are limited data comparing different treatment strategies in PLWH with a late HIV diagnosis, largely because of the tendency to exclude individuals with active AIDS-defining illnesses from phase 3 ART randomized clinical trials (RCTs). However, subgroup analyses from registration RCTs for modern ART regimens either do not indicate differences in virologic suppression rates between active and control study arms when data are stratified by CD4 cell count [22–26], or differences are not related to virological factors [27]. It should, however, be noted that numbers of individuals with low CD4 cell counts in such trials are limited. Prospective studies conducted specifically in individuals with advanced HIV disease may also struggle to recruit the planned numbers of participants, potentially due to high internalized stigma in PLWH who are diagnosed late; this restriction limits the statistical power of such trials [28]. However, some prospective clinical trials have studied PLWH diagnosed with advanced HIV disease. An RCT investigating raltegravir-intensified ART in advanced HIV disease in Africa (*N* = 1805) showed that participants with raltegravir-intensified ART had significantly greater initial viral load suppression compared with those receiving standard ART (week 4: 41.0% vs 13.4%; week 12: 71.9% vs 51.7%) (both *p* < 0.001). However, no discernable clinical benefit was observed at week 48 with intensified treatment, with similar mortality rates observed between treatment groups. Interestingly, there was no evidence of an increase in the rate of immune reconstitution inflammatory syndrome in participants receiving the integrase (INSTI)-boosted regimen (compared with standard ART) at week 48, suggesting that INSTIs can be used safely as part of initial ART regimens in patients with advanced HIV disease [29]. More recently, similar results were also shown when comparing INSTI- and PI-based regimens in a European cohort of patients with advanced HIV disease [30]. The REALITY and IDEAL studies (in participants with severe immunodeficiency [< 100 cells/mL] or presenting with acute AIDS-defining events, respectively) showed that immediate initiation of ART in conjunction with treatment for an OI did not compromise viral suppression or safety outcomes/quality of life measures [31, 32]. A study by Brites et al*.* (published in 2018) compared raltegravir with lopinavir/ritonavir for the treatment of pregnant women with a late HIV diagnosis. Virological suppression at delivery was achieved in 76.5% (13/17) of patients in the raltegravir group, versus 25.0% (4/16) of patients in the lopinavir/ritonavir group (relative risk: 3.1; 95% confidence interval [CI]: 1.3–7.4), with the authors proposing that the INSTI may be a suitable first-line option for pregnant women [33].

Owing to the complexities of recruiting patients with late HIV diagnoses, retrospective cohort study designs are more often used to study this patient group. In a 2019 study by Gianotti et al. boosted protease inhibitor- (PI; 696 patients), non-nucleotide reverse transcriptase inhibitor- (NNRTI; 184 patients [94% received efavirenz]), and INSTI-based (315 patients) regimens were compared in patients with advanced disease (defined as a CD4 count < 200 cells/µL and HIV-RNA > 5 log_10_ copies/mL). The primary endpoint was treatment failure (a composite endpoint defined as virological failure [first of two consecutive HIV-RNA > 50 copies/mL after 6 months of treatment]), discontinuation, or death. This study showed that the durability of INSTI-based regimens was higher than that of both the NNRTI- and boosted PI-based regimens. INSTI-based regimens were also associated with lower probabilities of virological failure and of discontinuation due to intolerance or toxicity [34]. A multi-center study published in 2021 compared rates of discontinuation and virological response in participants with a CD4 count < 200 cells/µL, who received either an INSTI-based or PI-based ART regimen as first-line therapy in Germany (*N* = 218) [30]. No statistically significant differences in treatment discontinuation due to adverse events (AEs), virological response (< 50 HIV-RNA copies/mL), or mortality were observed between treatment groups at weeks 12 or 48 [30]. There was, however, a trend toward higher rates of discontinuation due to an AE at week 48 with PI-based (18.2% [6/33]) versus INSTI-based (7.1% [3/42]) treatment regimens [30]. A much larger study published the same year by Rava et al. (*N* = 8002; 48.7% of whom had a late HIV diagnosis), compared discontinuation rates and virological and immunological response rates among groups of patients from the Cohort of the Spanish HIV/AIDS Research Network, who were receiving either INSTI-, NNRTI-, or PI-based regimens as first-line treatment for HIV [35]. At week 48, those diagnosed late had similar viral suppression, but worse immunological response, than those not diagnosed late [35]. Patients with a late diagnosis who were receiving NNRTI-based regimens had a higher rate of viral suppression (85.8%) than those on INSTI-based regimens (83.2%) (adjusted OR 1.36; 95% CI: 1.00–1.85), driven by better viral suppression with tenofovir disoproxil (TDF)/emtricitabine (FTC)/rilpivirine versus abacavir/lamivudine (3TC)/dolutegravir (DTG) [35]. Similar immunological response rates were observed across the different treatment groups; a significantly lower rate of discontinuations due to AEs was seen for INSTI-based regimens (5.6% [44/792]) versus NNRTI-based (10.8% [192/1786]) and PI-based (11.4% [151/1322]) regimens [35].

Of note, these studies pre-date the availability of the PI-based STR darunavir/cobicistat /FTC/tenofovir alafenamide (TAF) and the INSTI-based STR TAF/FTC/bictegravir, which were approved as first-line treatments in Europe in 2017 and 2018, respectively [36, 37]. Both are listed among the recommended treatment options in general, and rapid start specifically, in EACS and German–Austrian guidelines and might therefore be among those regimens frequently selected for treatment in situations of late diagnosis [18, 19]. The comparative efficacy and safety of these newer regimens in patients being diagnosed late for treatment are currently being compared in The Late Presenter Treatment Optimisation (LAPTOP) randomized, open-label trial (NCT03696160), which is the first study of this kind to be conducted specifically in this patient group. Results from studies such as this will help to inform treatment of HIV in this population with unique medical challenges.

## Recent and emerging challenges for late HIV diagnosis in Germany

### Impacts of the global COVID-19 pandemic on late HIV diagnosis

Data are now starting to emerge on the impacts of the COVID-19 pandemic on late HIV diagnosis. Given the global nature of this event, and potential insights that may be gained by looking at its effects on late HIV diagnoses in different healthcare systems, relevant studies conducted outside of Germany are also reviewed in this section.

The absolute number of people with advanced HIV disease at diagnosis decreased in all patient groups (heterosexuals, intravenous drug users, and MSM) in Germany from 2019 to 2021 (and in 2020 in particular), according to RKI data [5, 38]. However, the proportion of individuals presenting with a late diagnosis of HIV increased during this period, probably due to the significant reduction in the total number of new HIV diagnoses recorded during this time (the pandemic reduced the opportunity for both sexual contact and access to routine testing) [5, 38]. A single-center retrospective cohort study at a specialized center in Germany reported an increase in the rate of late HIV diagnoses during the pandemic (83%; 34/41) compared with that seen pre-pandemic (59%; 20/34); increased incidences of late diagnosis (*p* = 0.020) and lower CD4 counts (< 350 cells/µL [*p* = 0.037]; < 200 cells/µL [*p* = 0.022]) were associated with the ongoing COVID-19 pandemic in this study [39]. A recent study conducted in the Netherlands reported lower mean CD4 values in individuals diagnosed after lockdown compared with those diagnosed during lockdown (290 vs 420 cells/µL respectively), further highlighting the potential effects of the pandemic on the clinical presentation of late HIV diagnoses [40].

An Italian multicenter observational study noted a significant and disproportionate decrease in the rate of new diagnoses occurring among MSM during the COVID-19 pandemic compared with the preceding period [41]. Interestingly, this study also found that individuals diagnosed with HIV during versus before the pandemic were older (39.9 vs 34.0 years) with significantly higher median CD4 counts (305 vs 205 cells/µL), translating into a significant overall reduction in the prevalence of late HIV diagnosis (57.4% vs 63.6%) [41]. This was attributed to routine testing for HIV in individuals with suspected COVID-19, with the unanticipated consequence of catching HIV earlier in older individuals, who are usually harder to reach than younger people [41].

Interestingly, in a collaborative testing program involving 13 healthcare centers on the South and West Sides of Chicago (USA), there was an increase in the rate of diagnosis of acute HIV infection through routine ED screening of individuals with an influenza-like illness during the COVID-19 pandemic [42]. Conversely, the pandemic may have also exacerbated missed opportunities for the early detection of HIV because symptoms of acute HIV infection (e.g., fever, fatigue, and sore throat) may have been mistakenly attributed to COVID-19 [43, 44]. Indeed, physicians need to be aware of the risks and symptoms of acute HIV infection and include HIV testing in any person who presents with signs and symptoms suggestive of COVID-19, influenza, or other viral syndromes [45, 46].

### Emerging impacts of the Ukraine war on late diagnosis in Germany

**Fig. 5:**
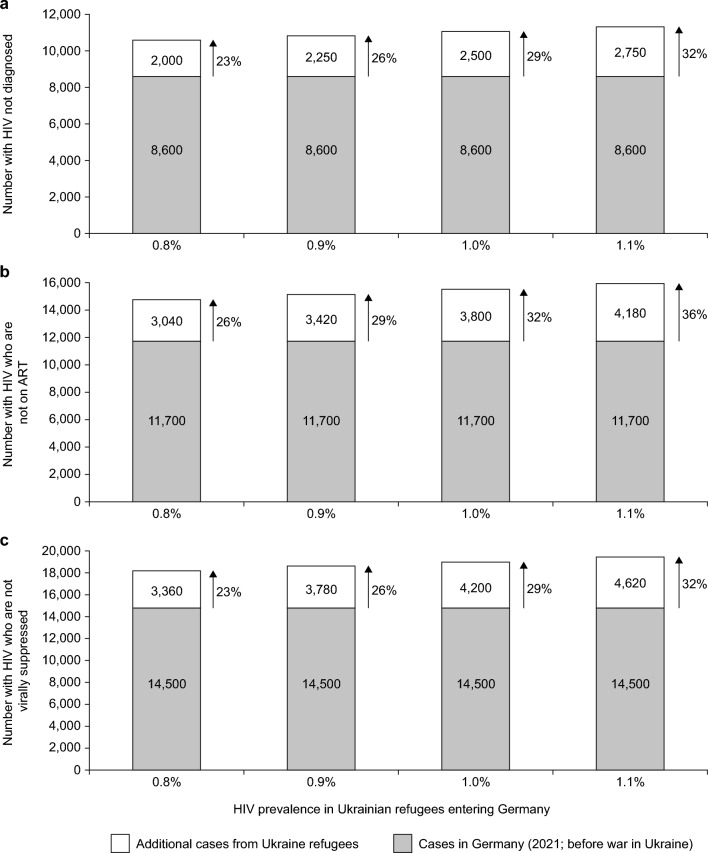
Potential impacts of the Ukraine war on rates of undiagnosed HIV in Germany. Estimates are based on the assumption of 1,000,000 Ukrainian refugees entering Germany, continuum of care estimates for the prevalence of HIV and HIV status (i.e., diagnosed, under ART, virally suppressed) in Ukraine (2022) [47, 48], and data from the RKI HIV in Germany 2021 epidemiological bulletin on the prevalence of HIV and HIV status in Germany [5]. *HIV* human immunodeficiency virus, *RKI* Robert Koch Institute

The HIV epidemic in Ukraine is among the worst in Europe, with a prevalence (range) of 0.9 (0.8–1.1)% and 240,000 people infected [47]. Continuum of care estimates for 2022 report that 75% of people with HIV from Ukraine are aware of their status, 62% are receiving ART, and only 58% achieve virologic suppression [47, 48]. At the time of writing, over 1,000,000 refugees from the Ukraine war are estimated to have entered Germany [49]. Assuming a prevalence rate of 1%, this would result in an additional 10,000 people with HIV living in Germany; of these, 25% (2500 people) would not be aware of their status, 38% (3800 people) would not be on treatment, and 42% (4200 people) would not be virally suppressed. As shown in Fig. 5 (which considers the full prevalence range), the influx of these refugees could potentially add significant pressure to each stage of the treatment cascade in Germany.

The absolute number of undiagnosed HIV cases in Germany (estimated at 8600 in 2021) [5] could be expected to have increased by 2000−2750 (23−32% vs 2021), with similar increases in both the number of people with HIV who are not on ART (3040−4180; 26−36% increase) and those not virally suppressed (3360−4620; 23−32% increase). Due to the significant challenges associated with migration (discussed in more detail below), it is unlikely the continuum of care estimates for Ukraine will be maintained in the refugee population in Germany, meaning the estimates shown in Fig. 5 may be conservative. However, this may be countered by the higher proportion of women and children in the refugee population, who report a lower prevalence of HIV compared with men. Indeed, numbers in Fig. 5 are merely estimates; the true impact of the Ukraine war on late diagnosis in Germany will be known only with time.

The German Federal government has reacted to this healthcare crisis by allowing Ukrainian refugees access to the national health insurance system, a prerequisite to continuing or linking to care [50–53]. However, although Germany (especially Berlin [54]) has been extremely successful at linking patients to care once HIV is diagnosed, this may be challenging for Ukrainian refugees with no experience of the German healthcare system. Indeed, in contrast to Germany, ART in Ukraine is (anecdotally) available over the counter without prescription [55]. Therefore, even among individuals who have previously presented with HIV in Ukraine and received ART, there is a substantial risk of being lost to care because of difficulties navigating the foreign German healthcare system while experiencing significant refugee trauma and engrained HIV stigma [56–58]. Not only does this pose an individual risk of disease progression, but it also has the potential to increase rates of transmission. Healthcare professionals in Germany may also face challenges with continuation of care for refugees who have been prescribed regimens that are not available as STRs in Germany (such as ‘TLD’, a fixed-dose combination of TDF, 3TC, and DTG). In such cases, a treatment change to a multi-tablet regimen with the same active substance or another STR with a different active substance would be necessary. In addition, a lack of individual treatment awareness and depleted stocks of ART on arrival add to the difficulties in managing the re-diagnosis of such patients.

### Migrants without health insurance in Germany

Lack of health insurance has long been reported as a barrier to HIV treatment [59], and continues to have a negative impact on entry into care for migrants from low- and middle-income countries living in high-income countries [60]. Migrant populations enter Germany from both the European Union (EU) and non-EU countries, with migrants from sub-Saharan Africa accounting for 10–15% of all new HIV diagnoses in Germany [61, 62]. A survey on the impact of health insurance status on access to healthcare and HIV testing among migrants from sub-Saharan Africa in Germany (2015) showed that having no health insurance or medical treatment voucher decreased the odds of contact with the healthcare system more than other socio-demographic characteristics (adjusted OR 0.36; 95% CI: 0.21–0.60; *p* < 0.001) [63]. In addition, migrants without health insurance were less likely to have ever received an HIV test compared with participants with health insurance (adjusted OR 0.55; 95% CI: 0.31–0.95; *p* = 0.033) [63]. In a qualitative study across nine German cities, 12 experts from local and state-level public health authorities and social security offices were interviewed (between July 2017 and January 2018) to assess their work with uninsured migrants [64]. The main challenge reported for chronic diseases (HIV, hepatitis and diabetes) was the cost associated with regular diagnostics and medications, making the provision of healthcare to uninsured migrants particularly challenging. Considering the disproportionate representation of migrants among those diagnosed with HIV in Germany and the challenges in providing treatment, this continues to be a significant challenge for patients, healthcare providers and the frameworks in which they exist.

## Overcoming barriers to early HIV diagnosis in Germany

The 2010 European Centre for Disease Prevention and Control (ECDC) guidelines recommend normalizing HIV testing in general settings, noting that this is the key to ending HIV transmission [65, 66]. A 2019 systematic review (carried out to inform future ECDC guidelines) analyzed the uptake and coverage of HIV testing outside of healthcare settings in the EU and showed this to be a widely accepted and effective way at reaching people who had never been tested for HIV [67]. In Germany, patient-initiated HIV testing has worked well (particularly among MSM), with over 90% of patients estimated to be aware of their HIV status [5]. This may be due to the ease of access to self-tests in Germany, the anonymity of testing possible in a range of settings (including AIDS Service Organizations, public health departments, and specialized health centers/physicians), and the fact there is no cost for an HIV test if it is clinically indicated. The increasing number of ‘higher-risk’ individuals accessing PrEP (and therefore being tested regularly) might also help to identify those who are unknowingly infected [19]. However, uptake and successful implementation of other approaches (such as indicator testing and universal ED testing) remain barriers to early HIV diagnosis in Germany.

### Indicator HIV testing

Provider-initiated indicator testing is key to identifying individuals who are at the greatest risk of late diagnosis in Germany, namely older patients, heterosexuals, and migrants [5, 12, 14, 16]. However, there is a lack of robust systems for provider-initiated testing (either routinely or based on ICs) and a finite capacity of primary care providers to apply such strategies in the face of more prevalent/higher-priority diseases. Many individuals who are diagnosed late with HIV in Germany have prior contact with the healthcare system with an IC but do not undergo HIV testing, with women and heterosexual men with HIV at particularly high risk of not being diagnosed despite having an IC [16]. This highlights a significant missed opportunity for early diagnosis. The FindHIV study aims to address this issue by developing a scoring system that can be applied by primary care physicians to trigger HIV testing based on an unbiased assessment of patient risk [68, 69]. This scoring system will be built on a comprehensive assessment of the patient characteristics that are associated with late diagnosis in Germany [68]. The FindHIV initiative will be a crucial tool for reducing rates of late HIV diagnosis in Germany. Other initiatives are, however, also required.

### Universal HIV testing

Limitations of indicator testing and the lack of any previous healthcare consultation before late HIV (or AIDS) diagnosis in many individuals mean that universal HIV testing should also be used to address the problem of late diagnosis. Opt-out testing in EDs, whereby HIV testing is carried out unless patients choose to decline, offers a range of benefits that are conducive to earlier detection of HIV. There is a greater prevalence of HIV in EDs versus other settings, meaning that the ED could catch undiagnosed people with HIV who are unlikely to be screened elsewhere [70–72]. Universal ED testing may also reduce the stigma associated with HIV testing, provide greater ease of automation within the hospital setting, and remove the need for physicians to remember to consult for HIV testing. Universal ED testing has shown benefits in terms of re-linking patients with HIV to care who had previously been lost to care [70, 71, 73], and may also improve early detection in individuals who are less likely to present to primary care (such as Ukrainian refugees and migrants without health insurance, as discussed above). An oft-stated concern around universal ED testing is that it may place an undue burden on healthcare professionals. However, numerous studies have demonstrated the feasibility and effectiveness of integrating universal testing (both for HIV and hepatitis B/C) into this setting, provided simple methods and training are available for establishing these processes [73–78]. Most recently, results from the first three years of an opt-out HIV testing program in the UK (using standard biochemistry samples taken in the ED) has shown that this method is feasible and acceptable, with relatively little disruption to standard ED protocol [79].

A 2018 systematic review by Bert et al. on the cost-effectiveness of routine HIV screening in high-income countries showed the most cost-effective option would be a one-time testing of the general population, with annual screening of high-risk groups. In this study, EDs, primary care, sexually transmitted disease clinics, and substance abuse treatment programs were identified as the most useful settings for such testing [80]. However, a certain background HIV prevalence might be needed to generate cost-effectiveness for universal testing, as has been shown for IC-guided HIV testing in the HIV Indicator Diseases across Europe Study, in which an undiagnosed HIV prevalence of at least 0.1% was required [80, 81].

**Fig. 6:**
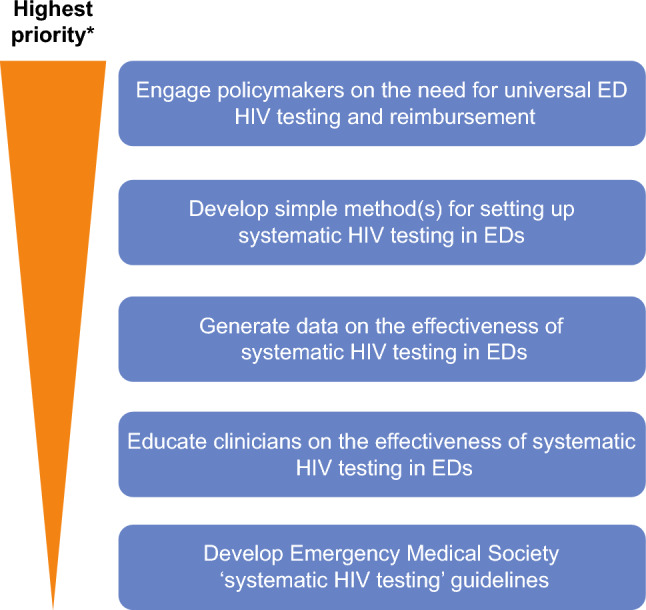
Actions required to advance HIV testing in EDs in Germany. *All listed activities should be considered essential. *ED* emergency department, *HIV* human immunodeficiency virus

In December 2021, the UK Government committed £20,000,000 to fund opt-out HIV testing at EDs in three large metropolitan areas, which together account for at least a third of undiagnosed cases [82]. Through such initiatives (and in line with current guidelines [83]) the UK aims to reduce the proportion of undiagnosed people with HIV below current levels (6% in 2019) [84], although there are concerns that the implementation is not more widespread [82]. Real-world data such as these will provide additional information on the practicalities and effectiveness of universal ED testing for HIV and guide future testing recommendations. These recommendations would form part of the multi-layered approach required to establish and advance successful universal testing in EDs in Germany. Suggested key actions for this implementation are presented in Fig. 6, with the highest priority being engagement with policymakers to convince them of the need for universal ED HIV testing and reimbursement.

## Conclusions and a call to action

Although treatment success in PLWH with late diagnosis is high, especially with robust ART options, late HIV diagnosis is nevertheless associated with increased mortality and morbidity. The consistently high rate of late diagnosis in Germany confirms the need for continued efforts to reduce these numbers. Missed opportunities in the healthcare system to identify those who are unknowingly infected with HIV seem to pose a significant challenge in Germany. The COVID-19 pandemic and the ongoing war in Ukraine have both challenged the HIV response in Germany, but in doing so have identified key areas needing additional support. The issue of late diagnosis does not affect all patient populations equally; identifying those patients at risk of late diagnosis will therefore require a more nuanced approach. A universal ED testing strategy seems like an efficient use of healthcare resources and would provide a major tool for tackling late HIV diagnosis in Germany and managing undiagnosed HIV among those less likely to present to primary care centers. It could also be beneficial in re-linking patients with HIV to care who may have been lost during the COVID pandemic or as a result of migration. However, acceptance of the need for additional testing and prevention strategies still needs to be built in Germany, as exemplified by the fact that only two German cities (Berlin and Bochum) have registered as fast-track cities with a commitment to initiatives aimed at ending the global HIV epidemic [2]. This possibly reflects the somewhat conservative German political landscape, and perceptions that undiagnosed HIV is not a relevant problem. Safety and efficacy results from trials such as the LAPTOP study using the most up-to-date HIV regimens should help to raise awareness of this unique patient group and guide strategies to help earlier diagnosis.

In conclusion, combined efforts are needed to reduce the persistent challenge of late diagnosis in Germany, including greater political will, increased funding of education and testing campaigns by governmental institutions, continued education about HIV testing by HIV experts and pharmaceutical companies, and broad testing support by physicians who are not routinely involved in HIV care. This approach is necessary to ensure that Germany can meet the UNAIDS 95-95-95 aims by 2030.

## Data Availability

Not applicable.
